# Supplementary material for: Comparing Predictive Utility of Head Computed Tomography Scan-Based Scoring Systems for Traumatic Brain Injury: A Retrospective Study
Source: Brain Sci. 2023 Jul 29;13(8):1145. doi: 10.3390/brainsci13081145 (PMC10452909; doi:10.3390/brainsci13081145)
Supplement: Supplementary file 1 [file brainsci-2518590-supplementary.pdf › brainsci-2518590-supplementary.pdf]

Supplementary Table S1. Distribution of patients in Marshall, Rotterdam, NIRIS, and Helsinki categories according to outcome variables

		In-hospital mortality (n = 996)			ICU admission (n = 994)[1]			Neurosurgery (n = 996)			THLOS ≥ seven days (n = 995)[2]		
		Yes (n=27)	No (n=969)	p	Yes (n=207)	No (n=787)	p	Yes (n=82)	No (n=914)	p	Yes (n=205)	No (n=790)	p
Marshall	I, n (%)	0 (0)	646 (66.7)	< 0.001	38 (18.4)	607 (77.1)	< 0.001	1 (1.2)	645 (70.6)	< 0.001	66 (32.2)	580 (73.4)	< 0.001
	II, n (%)	7 (25.9)	243 (25.1)		87 (42.0)	162 (20.6)		16 (19.5)	234 (25.6)		81 (39.5)	169 (21.4)	
	III, n (%)	6 (22.2)	39 (4.0)		33 (15.9)	12 (1.5)		22 (26.8)	23 (2.5)		28 (13.7)	17 (2.2)	
	IV, n (%)	3 (11.1)	2 (0.2)		4 (1.9)	1 (0.1)		4 (4.9)	1 (0.1)		1 (0.5)	3 (0.4)	
	V, n (%)	0 (0)	0 (0)		0 (0)	0 (0)		0 (0)	0 (0)		0 (0)	0 (0)	
	VI, n (%)	11 (40.7)	39 (4.0)		45 (21.7)	5 (0.6)		39 (47.6)	11 (1.2)		29 (14.1)	21 (2.7)	
Rotterdam	1, n (%)	0 (0)	39 (4.0)	< 0.001	14 (6.8)	25 (3.2)	< 0.001	4 (4.9)	35 (3.8)	< 0.001	9 (4.4)	30 (3.8)	< 0.001
	2, n (%)	3 (11.1)	766 (79.1)		80 (38.6)	687 (87.3)		17 (20.7)	752 (82.3)		101 (49.3)	668 (84.6)	
	3, n (%)	7 (25.9)	131 (13.5)		70 (33.8)	68 (8.6)		26 (31.7)	112 (12.3)		64 (31.2)	74 (9.4)	
	4, n (%)	6 (22.2)	19 (2.0)		20 (9.7)	5 (0.6)		17 (20.7)	8 (0.9)		14 (6.8)	11 (1.4)	
	5, n (%)	7 (25.9)	10 (1.0)		16 (7.7)	1 (0.1)		13 (15.9)	4 (0.4)		13 (6.3)	3 (0.4)	
	6, n (%)	4 (14.8)	4 (0.4)		7 (3.4)	1 (0.1)		5 (6.1)	3 (0.3)		4 (2.0)	4 (0.5)	
NIRIS	0, n (%)	0 (0)	577 (59.5)	< 0.001	35 (16.9)	541 (68.7)	< 0.001	1 (1.2)	576 (63.0)	< 0.001	62 (30.2)	515 (65.2)	< 0.001
	1, n (%)	0 (0)	167 (17.2)		24 (11.6)	143 (18.2)		0 (0)	167 (18.3)		28 (13.7)	139 (17.6)	
	2, n (%)	9 (33.3)	174 (18.0)		86 (41.5)	96 (12.2)		27 (32.9)	156 (17.1)		74 (36.1)	109 (13.8)	
	3, n (%)	8 (29.6)	26 (2.7)		33 (15.9)	1 (0.1)		27 (32.9)	7 (0.8)		21 (10.2)	13 (1.6)	
	4, n (%)	10 (37.0)	25 (2.6)		29 (14.0)	6 (0.8)		27 (32.9)	8 (0.9)		20 (9.8)	14 (1.8)	
Helsinki	-3, n (%)	0 (0)	43 (4.4)	< 0.001	12 (5.8)	30 (3.8)	< 0.001	3 (3.7)	40 (4.4)	< 0.001	8 (3.9)	35 (4.4)	< 0.001
	-2, n (%)	0 (0)	9 (0.9)		5 (2.4)	4 (0.5)		4 (4.9)	5 (0.5)		4 (2.0)	5 (0.6)	
	-1, n (%)	1 (3.7)	19 (2.0)		12 (5.8)	8 (1.0)		5 (6.1)	15 (1.6)		7 (3.4)	13 (1.6)	
	0, n (%)	1 (3.7)	742 (76.6)		71 (34.3)	671 (85.3)		10 (12.2)	733 (80.2)		97 (47.3)	646 (81.8)	
	1, n (%)	0 (0)	6 (0.6)		3 (1.4)	3 (0.4)		2 (2.4)	4 (0.4)		4 (2.0)	2 (0.3)	
	2, n (%)	3 (11.1)	86 (8.9)		30 (14.5)	59 (7.5)		8 (9.8)	81 (8.9)		27 (13.2)	62 (7.8)	
	3, n (%)	6 (22.2)	24 (2.5)		23 (11.1)	7 (0.9)		11 (13.4)	19 (2.1)		19 (9.3)	10 (1.3)	
	4, n (%)	2 (7.4)	12 (1.2)		12 (5.8)	2 (0.3)		10 (12.2)	4 (0.4)		10 (4.9)	4 (0.5)	
	5, n (%)	0 (0)	11 (1.1)		9 (4.3)	2 (0.3)		7 (8.5)	4 (0.4)		8 (3.9)	3 (0.4)	
	6, n (%)	2 (7.4)	2 (0.2)		4 (1.9)	0 (0)		4 (4.9)	0 (0)		2 (1.0)	2 (0.3)	
	7, n (%)	5 (18.5	6 (0.6)		11 (5.3)	0 (0)		9 (11.0)	2 (0.2)		8 (3.9)	3 (0.4)	
	8, n (%)	1 (3.7)	2 (0.2)		3 (1.4)	0 (0)		2 (2.4)	1 (0.1)		1 (0.5)	2 (0.3)	
	9, n (%)	2 (7.4)	5 (0.5)		7 (3.4)	0 (0)		4 (4.9)	3 (0.3)		6 (2.9)	1 (0.1)	
	10, n (%)	1 (3.7)	0 (0)		1 (0.5)	0 (0)		0 (0)	1 (0.1)		1 (0.5)	0 (0)	
	11, n (%)	1 (3.7)	2 (0.2)		2 (1.0)	1 (0.1)		3 (3.7)	0 (0)		2 (1.0)	1 (0.1)	
	12, n (%)	2 (7.4)	0 (0)		2 (1.0)	0 (0)		2 (0.2)	0 (0)		1 (0.5)	1 (0.1)	
	13, n (%)	0 (0)	0 (0)		0 (0)	0 (0)		0 (0)	0 (0)		0 (0)	0 (0)	
	14, n (%)	0 (0)	0 (0)		0 (0)	0 (0)		0 (0)	0 (0)		0 (0)	0 (0)	

1. Two patients had unknown ICU admission status.
2. One patient had unknown THLOS.

CT: computed tomography; ICU: intensive care unit; IQR: interquartile range; NIRIS: NeuroImaging Radiological Interpretation System; THLOS: total hospital length of stay
